# Generation of megabase-scale deletions, inversions and duplications involving the *Contactin-6* gene in mice by CRISPR/Cas9 technology

**DOI:** 10.1186/s12863-017-0582-7

**Published:** 2017-12-28

**Authors:** Alexei N. Korablev, Irina A. Serova, Oleg L. Serov

**Affiliations:** 1 Department of Molecular Mechanisms of Development, Institute of Cytology and Genetics, Russia Academy of Sciences, Siberian Branch, Novosibirsk, 630090 Russia; 2 Research Institute of Medical Genetics, Tomsk National Research Medical Center Russian Academy of Sciences, Tomsk, 634050 Russia

**Keywords:** CRISPR/Cas9 technology, Megabase-scale deletions, Duplications and inversions, *Cntn6* gene, Cytoplasmic microinjections into mouse zygotes

## Abstract

**Background:**

Copy Number Variation (CNV) of the human *CNTN6* gene (encoding the contactin-6 protein), caused by deletions or duplications, is responsible for severe neurodevelopmental impairments, often in combination with facial dysmorphias. Conversely, deleterious point mutations of this gene do not show any clinical phenotypes. The aim of this study is to generate mice carrying large deletions, duplications and inversions involving the *Cntn6* gene as a new experimental model to study CNV of the human *CNTN6* locus.

**Results:**

To generate large chromosomal rearrangements on mouse chromosome 6, we applied CRISPR/Cas9 technology in zygotes. Two guide RNAs (gRNAs) (flanking a DNA fragment of 1137 Mb) together with Cas9 mRNA and single-stranded DNA oligonucleotides (ssODN) were microinjected into the cytoplasm of 599 zygotes of F1 (C57BL x CBA) mice, and 256 of them were transplanted into oviducts of CD-1 females. As a result, we observed the birth of 41 viable F0 offspring. Genotyping of these mice was performed by PCR analysis and sequencing of PCR products. Among the 41 F0 offspring, we identified seven mice with deletions, two animals carrying duplications of the gene and four carrying inversions. Interestingly, two F0 offspring had both deletions and duplications. It is important to note that while three of seven deletion carriers showed expected sequences at the new joint sites, in another three, we identified an absence of 1–10 nucleotides at the CRISPR/Cas9 cut sites, and in one animal, 103 bp were missing, presumably due to error-prone non-homologous end joining. In addition, we detected the absence of 5 and 13 nucleotides at these sites in two F0 duplication carriers. Similar sequence changes at CRISPR/Cas9 cut sites were observed at the right and left boundaries of inversions. Thus, megabase-scale deletions, duplications and inversions were identified in 11 F0 offspring among 41 analyzed, i.e., approximately 25% efficiency. All genetically modified F0 offspring were viable and able to transmit these large chromosomal rearrangements to the next generation.

**Conclusions:**

Using CRISPR/Cas9 technology, we created mice carrying megabase-scale deletions, duplications, and inversions involving the full-sized *Cntn6* gene. These mice became founders of new mouse lines, which may be more appropriate experimental models of CNV in the human 3p26.3 region than *Сntn6* knockout mice.

## Background

Peritelomeric region pter-p26.3 of human chromosome 3 contains three genes of the immunoglobulin superfamily: *CHL1*, encoding neural cell adhesion molecule L1-like protein, and both *CNTN4* and *CNTN6*, which encode contactin-4 and contactin-6 proteins, respectively. These genes are considered to be candidates for causing intellectual impairment based mainly on analysis of deletions and duplications in 3p26.3 [1–6]. In fact, copy number variations (CNV) in the 3p26.3 region, due to microdeletions and microduplications involving and one or more genes, are accompanied by severe neurodevelopmental disorders including intellectual disability, developmental delay, autism spectrum disorders, seizures and attention deficit hyperactivity [1–6]. Sizes of deletions and duplications in the 3p26.3 region vary from hundreds of kb to over three Mb. The *CNTN6* gene is often involved in CNV in the 3p26.3 region either alone or in combination with the *CHL1* and the *CNTN4* genes. For instance, among 14 patients carrying deletions (7 cases) or duplications (5 cases) involving only the *CNTN6* gene, eleven had neurodevelopmental disorders, and six had dysmorphic features [5]. In addition, two unrelated patients with various neurodevelopmental and neuropsychiatric disorders along with dysmorphic features were characterized; one had a microdeletion, and the other had a microduplication involving only the *CNTN6* gene [3]. Interestingly, pedigrees with inheritance of 3p26.3 microdeletions and microduplications involving the *CNTN6* gene include families with healthy or only mildly affected carriers in several generations [3, 5, 7].

The *CNTN6* gene is a member of the immunoglobulin superfamily of neural cell-adhesion molecules [8]. The *CNTN6* gene has a size of over 420 kB and contains 30 exons with sizes varying from 355 bp to 12,000 bp ([9]; https://www.ncbi.nlm.nih.gov/gene/27255). The CNTN6 protein promotes neurite outgrowth and synaptogenesis, especially in sensory-motor pathways and is crucial for the appropriate orientation of dendrite growth in cortical pyramidal neurons [10] and synapse formation in the cerebellum [11].

In contrast to the aforementioned severe neurodevelopmental disorders caused by CNV of the *CNTN6* gene, deleterious point mutations (missense, nonsense, splice site, or disrupted start or stop codon) of the *CNTN6* gene do not show any clinical phenotypes and have been revealed as rare mutations (approximately 1%) in a study of 942 healthy people [12]. In this instance, it is pertinent to note that knockout mice homozygous for *Cntn6* ^−/−^ are viable and fertile [13]. Moreover, the formation and organization of all nuclei and layers throughout the brains of these mutant mice appeared normal, but Cntn6 deficiency led to defects in motor coordination [13]. It is important to note that the *Cntn6*, *Chl1* and *Cntn4* genes are all located on mouse chromosome 6 at position 103,510,581–106,700,141 (GRCm38/mm10) and their order relative to each other in mice, *Chl1*, *Cntn6* and *Cntn4,* is the same as in humans.

The obvious contrast between the phenotypic manifestations of chromosomal rearrangements and point mutations at the *CNTN6* locus, in both humans and mice, prompted us to generate mice carrying megabase-scale deletions, duplications and inversions involving the *Cntn6* gene as a more appropriate experimental model for studying its CNV then knockout mutants.

## Results

**Fig. 1:**
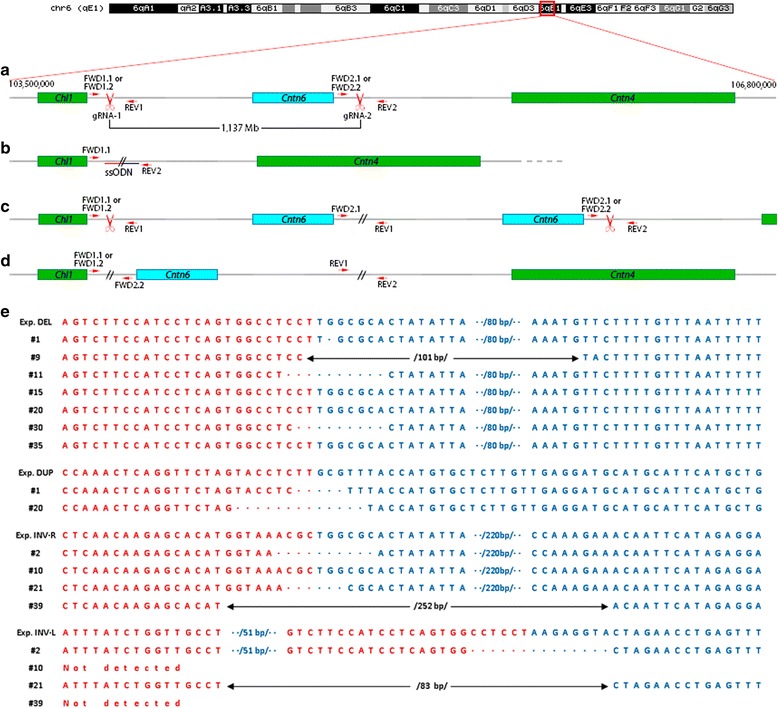
Scheme of targeted locus in wild-type and genome modified animals. **a** Ideogram of mouse chromosome 6 and detailed organization of the region limited by 103,500,000 and 106,800,000 positions which contains *Chl1*, *Cntn6* and *Cntn4* genes. Red arrows indicate positions sites for primers: FWD1.1, FWD1.2, REV1, FWD2.1 or FWD2.2, and REV2. These sites mark the boundaries of the presumable 1137 Mb deletion. **b** Genotyping of a deletion involving the *Cntn6* gene by primers FWD1.1 and REV2. Left and right shoulders of ssODN are shown red and blue, respectively. **c** Genotyping of duplication involving the *Cntn6* gene by primers FWD2.1 and REV1. **d** Genotyping of inversion involving the *Cntn6* gene by two pairs of primers: FWD1.1 or FWD1.2 (FWD1.2 was used only for prepare PCR-product for sequencing) and FWD2.2 for left side, and REV1 and REV2 for right side, respectively. **e** Exp Del, Exp Dup, Exp Inv-L and Exp Inv-R are expected sequences in new joint sites for deletion, duplication and inversions, respectively, due to correct reparation after Cas9-nuclease digest between 3rd and 4th nucleotides after PAM. Deletion junction sequences of 7 founders, #1, #9, #11, #15, #20, #30 and #35; duplication junction sequences of two founders, # 1 and #20; INV-R (right) junction sequences of four founders: #2, #10, # 21, and #39; INV-L (left) junction sequences of four founders: #2, #10, # 21, and #39

For generation of megabase-scale chromosome modifications involving the *Cntn6* gene, we used a recently proposed approach based on CRISPR/Cas9 technology [14]. Our scheme for generating deletions, duplications and inversions involving the *Cntn6* gene on mouse chromosome 6 is presented in Fig. 1a. Target sites for gRNAs were selected, flanking the DNA fragment containing the *Cntn6* gene (Table 1; Fig. 1a) based on the frequent occurrence of CNV in the homologous region of the human genome, 3p26.3 [2, 5]. According to Fig. 1a, this 1137 Mb DNA fragment includes the entire *Cntn6* gene with more than 0.5 Mb sequence upstream and approximately 100 kb downstream.

**Table 1:** Sequences of gRNAs complementary to target sites flanking the *Cntn6* gene and ssODN

gRNA	Sequences (5′ - 3′)	Target positions for gRNAs
gRNA-1	GAGCACATGGTAAACGCAGG(AGG)	chr6: 103,842,565-103,842,587: «-»
gRNA-2	GGTAATATAGTGCGCCAAAG(AGG)	chr6: 104,979,795-104,979,817: «-»
ssODN	5’GGTCCTTGGAGAGGAACATGGCCTTGCTTCTGTGCAGTCTTCCATCCTCAGTGGCCTCCTTGGCGCACTATATTACCTAATCCATTTTTCAGCATCCAAGATATTTTAAAGAAGAAGAAA3’ « + »	

We injected a pair of gRNAs together with Cas9 mRNA and ssODN into the cytoplasm of 599 zygotes and subsequently transplanted 256 viable 1-cell embryos into 11 recipient CD-1 females at a rate of 10–17 zygotes per female (Table 2). Birth of 41 live offspring was observed from 10 recipient females, on average 4–6 offspring per female, excluding one that did not give offspring and one instance of stillbirth (Table 2). Three offspring died shortly after birth (Table 2).

**Table 2:** Manipulations of zygotes and birth efficiency of F0 offspring with genetic modifications: deletions, duplications and inversions

Number of zygotes microinjected	Number of transplanted zygotes	Number of recipient females	Number of mice born alive	Number of mice born dead	Number of F0 offspring with deletions	Number of F0 offspring with inversions	Number of F0 offspring with duplications
599	256	11	41	4	7	4	2

**Fig. 2:**
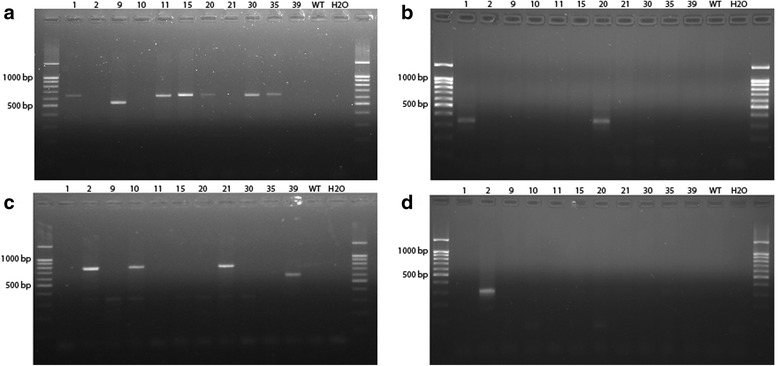
Results of genotyping of 11 offspring F0 by PCR for detection of carriers: deletion of the *Cntn6* gene (panel **a**), its duplications (panel **b**), its inversions sequenced right and left sides (panel **c** and **d**, respectively). Primer pairs used for detection of deletions were FWD1.1 + REV2, duplications were FWD2.1 + REV1 and inversions were REV1 + REV2 for right side, and FWD1.1 + FWD2.2 for left side. Expected size of PRC products: deletion 636 bp, duplication 349 bp, inversion right side 790 bp, inversion left side 323 bp

Genotyping of the 41 viable F0 offspring by PCR analysis (primers listed in Table 3) and subsequent PCR product sequencing allowed us to identify 11 offspring carrying genetic modifications such as deletions (Fig. 2a), duplications (Fig. 2b) and inversions (Fig. 2c, d) involving the *Cntn6* gene (Table 2). Figure 1e illustrates genomic DNA sequences from these 11 animals across the gRNA target sites. The data show that among seven animals carrying deletions, F0 offspring #15, #20 and #35 had expected deletion junction sequences (Fig. 1b) whereas heterogeneity was observed in another four offspring due to error-prone non-homologous end joining (Fig. 1e). Interestingly, F0 offspring #1 and #20 had both deletions and duplications (Fig. 1e) although their duplications lacked 5 and 13 nucleotides at the CRISPR/Cas9 cut sites, respectively.

**Table 3:** Primers used for genotyping F0 offspring with deletions, duplications and inversions

Primers	Sequence (5′ - 3′)	Positions on mouse chromosome 6
FWD1.1	TGGGTCCTTGGAGAGGAACA	chr6: 103,842,509-103,842,528
FWD1.2	ACTCTGGTGACAATGTGCGT	chr6: 103,841,824-103,841,843
REV1	TGCACATGACCCATGACCTC	chr6: 103,842,786-103,842,767
FWD2.1	TCCCCATCTGCTGGCTCTAT	chr6: 104,979,678-104,979,697
FWD2.2	AGAGGTTGATGCAAGCTGCC	chr6: 104,979,540-104,979,559
REV2	CCCCCAAGTGATGCTTCTGT	chr6: 104,980,355-104,980,374
FWD-SNP	TTGCCCTGGTTGTCTTTTATTCAT	chr6: 104,847,831-104,847,854
REV-SNP	AGCACAAACCATGTCACCAAG	chr6: 104,848,314-104,848,334

As is illustrated in Figs. 1e, 2c and d, F0 offspring #2, #10, #21 and #39 had inversions. It was possible to identify the precise right boundaries of all inversions whereas the left boundary was identified for #2 and #21.

It is important to note that in the one stillborn offspring and the three that died shortly after birth, there were no signs of sequence changes at the CRISPR/Cas9 cut sites. All eleven genetically modified F0 offspring were viable without visible anomalies. Six F0 males and four F0 females were crossed with C57BL and we observed birth of F1 offspring, on average nine offspring per female. These data imply that most F0 males and females were fertile.

**Fig. 3:**
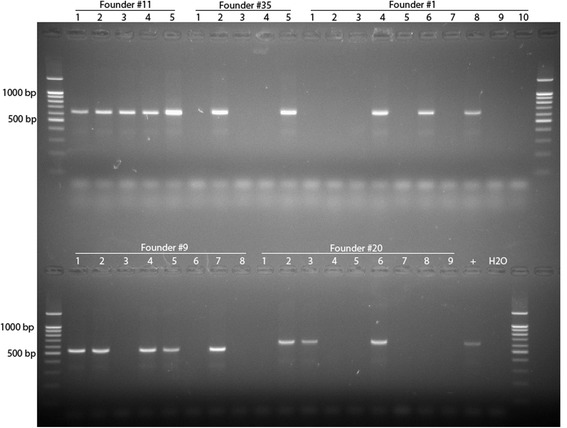
Results of genotyping of F1 mice (founders #11, #35, #1, #9 and #20) by PCR for detection of deletion of the *Cntn6* gene. Primer pairs used for detection of deletions were FWD1.1 + REV2

**Fig. 4:**
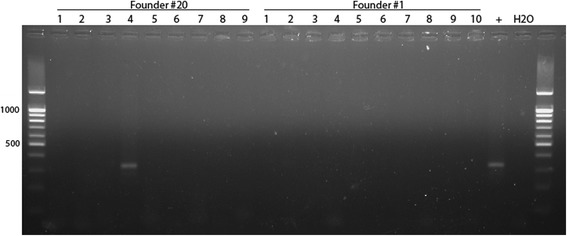
Results of genotyping of F1 mice (founders #20 and #1) by PCR for detection of duplication of the *Cntn6* gene. Primer pairs used for detection of duplication were FWD2.1 + REV1

**Fig. 5:**
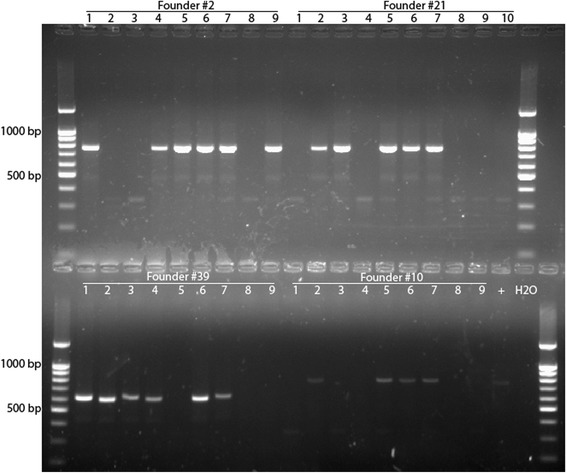
Results of genotyping of F1 mice (founders #2, #21, #39 and #10) by PCR for detection of inversions (right side only). Primer pairs used for detection inversions were REV1 + REV2 (for right side)

Figures 3, 4 and 5 present results of genotyping of F1 offspring derived of founders carrying deletions, duplications and inversions, respectively. As follows from the Figures, inheritance of induced deletions among F1 offspring from founders #35 and #9 is close to expected (Fig. 3). Similar situation with inheritance of inversions among F1 offspring from founders #21 and #10 (Fig. 5). However, notable deviations in heritance of deletion from the expected we observed among F1 offspring from founder #11 since all offspring was positive for deletion (Fig. 3). PCR analysis with using primers to exon 20 and both borders of the *Cntn6* gene (Table 3) demonstrated that founder F0 #11 was positive for presence of the sequences that means mosaicism, i.e., presence of two types of cells carrying either deletion or wild-type allele.

Interestingly, among 9 F1 offspring from founder #20 was identified one animal carrying duplication, and none among 10 F1 offspring from founder #1 (Fig. 4). Analysis of SNP (single nucleotide polymorphism) distinguishing the parental alleles of the *Cntn6* gene in founder #20 and its F1 offspring (Fig. 4, #4) demonstrated that founder #20 had both SNP parental variants (**T** and **C** in position 104,848,175 derived from C57BL/6 and CBA, respectively) but F1 offspring (#4) had **T** only. It is evidence that duplication occurred from single allele derived from C57BL/6.

All F1 offspring derived from nine founders were healthy without visible anomalies.

## Discussion

Genomic anomalies at the submicroscopic level are often based on variability of DNA copy number in unstable genome regions [15]. CNVs are frequently represented by DNA fragments larger than 1 Mb [16], which arise due to deletions and duplications involving single or multiple genes. CNVs significantly to the variability of our genome and are often associated (up to 15%) with severe disorders [17, 18].

Considering the evolutionary conservation of syntenic gene groups between humans and mice, it is possible to create mouse models of chromosomal deletions and duplications responsible for human disorders. The large sizes of such chromosomal rearrangements have led to major difficulties in realizing this approach. To overcome this obstacle, a multi-step approach has been previously employed, based on insertion of Cre-loxP sites at desirable chromosome sites in embryonic stem (ES) cells followed by recombination between them [19, 20]. Application of CRISPR/Cas9 technology in ES cells has allowed the generation of large deletions, duplications, and inversions, and then mice carrying these rearrangements can be produced by chimera technology [21]. However, these approaches are time consuming and laborious.

Recently, two protocols have been published [14, 22] based on direct microinjection of CRISPR/Cas9 components into zygotes, resulting in the generation of large deletions, inversions, and duplications, and leading to birth of genetically modified mice. We applied this new approach to generate megabase-scale deletions, duplication, and inversions involving the *Cntn6* gene. The desired chromosomal rearrangements were generated at up to 25% efficiency, with eleven genetically modified animals among F0 41 total offspring. Among the eleven genetically modified offspring, seven animals harbored deletions and two were identified as simultaneous carriers of deletions and duplications. Interestingly, similar sized deletions at the tyrosinase gene locus (Tyr) obtained by the same approach [14] were not accompanied by duplications.

All genetically modified F0 animals were viable and transmitted deletions, duplications and inversions to the next generation; hence, they became founders of new mouse lines with potential for studying the effects of CNV of the *Cntn6* gene. More generally, the method used here permits rapid in vivo modeling of large chromosomal rearrangements in mice.

## Conclusions

Thus, we created mice carrying megabase-scale deletions, duplications, and inversions involving the full-sized *Cntn6* gene through using CRISPR/Cas9 technology. This approach have provided to generate mice potentially modeling of human diseases due to CNV in the human 3p26.3 region than *Сntn6* knockout mice.

## Methods

### Animal maintenance

C57BL/6 male and F1 (C57BL/6 x CBA) female mice were used in experiments with early embryos. Pseudopregnant female CD-1 mice were used as recipients for transplantation of microinjected zygotes into oviducts. Food and water were available for animals ad libitum.

### CRISPR/Cas9 target sites and construction of plasmid vectors

Target sites for CRISPR/Cas9 were identified using https://crispr.med.harvard.edu/ as described previously [23]. Selection of the target sites for CRISPR/Cas9 was based on analysis of published deletions and duplications involving the human *CNTN6* gene [2, 5]. Plasmid vectors gRNA-CNTN6 containing gRNA sequences were prepared via modification of pSpCas9(BB)-2A–GFP (PX458) (Addgene) according to protocol [24].

### Preparation of Cas9 mRNA, gRNA and ssODN for microinjection

To synthesize gRNAs, we first amplified corresponding gRNA sequence from the gRNA-CNTN6 plasmid. We introduced the T7 promoter sequence at the 5′-end of the forward primer, which resulted in generation of a PCR product containing the T7 promoter adjacent to the first nucleotide of the gRNA. This PCR product was extracted from agarose gel using a kit (Biosilica, RF), precipitated with ethanol, and then used for in vitro transcription reactions. In vitro transcription was performed with a MEGAshortscript™ T7 Transcription Kit (Ambion) according to manufacturer’s protocol. For purification of gRNAs, we used a MEGAclear™ Transcription Clean-Up Kit (Ambion) according to manufacturer’s protocol. We purchased GeneArt™ CRISPR Nuclease mRNA to use as Cas9 mRNA (Thermo Fisher Scientific, USA).

Single-stranded DNA oligonucleotides (ssODN) were 120 bp in length (DNA Synthesis, Moscow, RF) and positioned directly adjacent to the external gRNA site [25]. ssODN had two homologous shoulders of 60 bp each and both were complementary to the «-»-DNA strand whereas both gRNAs were complementary to the « + »-strand. ssODN sequence was determined based on the Cas9 cut site between the 3rd and 4th nucleotides after PAM.

### Obtaining fertilized zygotes by superovulation

Five-week-old F1 (C57BL/6 x CBA) females were super-ovulated by intraperitoneal injection of 7.5 IU of pregnant mare serum (PMSG) at 16.00 h, and 45 h later by 7.5 IU human chorionic gonadotrophin (hCG). The next morning the females were checked for the presence of a vaginal copulation plug.

Females were sacrificed by cervical dislocation [26]. Oviducts of the fertilized females were dissected, and fertilized eggs with cumulus cells were placed in M2 medium (Sigma Aldrich, USA) 21–22 h after hCG injection. The fertilized zygotes were treated with hyaluronidase (Sigma Aldrich, H4272) to release them from cumulus cells, and then were cultured prior to cytoplasmic injection in drops of M16 medium (Sigma Aldrich, M7292) under mineral oil (Sigma Aldrich, M8410) at 37 °C and an atmosphere of 5% CO_2_.

### Cytoplasmic injection into zygotes

50 ng/μL Cas9 mRNA, 25 ng/μL gRNA (each) and 100 ng/μL ssODN were mixed in RNase-free water, backfilled into an injection needle with positive balancing pressure (Transjector 5246, Eppendorf, Germany) and injected into the cytoplasm of zygotes. After injections, the embryos were cultured for 1 h in drops of M16 medium at 37 °C and an atmosphere of 5% CO_2_.

The viable microinjected zygotes were transplanted the same day into oviducts of pseudopregnant CD-1 females (0.5 days after coitus). Isoflurane inhalation anesthesia was applied in these experiments.

### Genotyping and identification of mutant F0 founders and its F1 offspring

Genomic DNA was isolated from tail tips by first placing them in 500 μl of Tail Lysis Buffer containing 100 mM NaCl, 10 mM Tris pH 8.0, 25 mM EDTA, 0.5% sodium dodecyl sulfate (SDS), 0.2 μg/μl Proteinase K, and then incubating them at 56 °C until completely dissolved. Lysates were treated by a standard phenol-chloroform extraction method and then DNA was repeatedly precipitated with ethanol. PCR was performed with 2 μl (20 ng) of genomic DNA in 25 μl of mixture containing 1× Taq AS buffer (67 mM Tris-HCl, pH 8.8; 16.6 mM (NH_4_)_2_SO_4_, 0.01% Tween-20) with 1.5 mM MgCl_2_, 0.2 mM of each deoxynucleotide (dATP, dCTP, dGTP, and dTTP), 0.4 mM of forward and reverse primers, and 1 U of Taq polymerase. The reaction was conducted under the following conditions: initial denaturation at 95 °C 3 min, 35 cycles (30 s at 95 °C, 30 s at 63 °C and 2 min at 72 °C), and a final incubation at 72 °C for 3 min. PCR products were analyzed by electrophoresis with 2% agarose gels in Tris-EDTA-acetate buffer and sequenced using the BigDye® Terminator v3.1 kit (Thermo Fisher Scientific, USA) on an Applied Biosystems 3500 Genetic Analyzer according to manufacturer’s recommendations.

A list of primers used for genotyping is given in the Table 3.
